# A Mixed-Method Study on the Assessment of Factors Influencing Nurses’ Provision of Spiritual Care

**DOI:** 10.3390/healthcare12080854

**Published:** 2024-04-18

**Authors:** Evangelos C. Fradelos, Victoria Alikari, Sofia Artemi, Evdokia Missouridou, Polyxeni Mangoulia, Maria Kyranou, Maria Saridi, Aikaterini Toska, Konstantinos Tsaras, Foteini Tzavella

**Affiliations:** 1 Laboratory of Clinical Nursing, Department of Nursing, University of Thessaly, 41500 Larissa, Greece; saridim@hotmail.com (M.S.); ktoska07@yahoo.gr (A.T.); 2 Department of Nursing, University of West Attica, 12244 Egaleo, Greece; vicalikari@uniwa.gr (V.A.); evdmisouridou@yahoo.gr (E.M.); 3 General Hospital “Elpis”, 11522 Athens, Greece; sofiaartemi1977@gmail.com; 4 Department of Nursing, University of Athens, 10682 Athens, Greece; pmango@nurs.uoa.gr; 5 Department of Nursing, Cyprus University of Technology, Limassol 3036, Cyprus; maria.kyranou@cut.ac.cy; 6 Department of Nursing, University of Thessaly, 41500 Larissa, Greece; ktsa@uth.gr; 7 Department of Nursing, University of Peloponnese, 22131 Tripoli, Greece; ftzavella@hotmail.com

**Keywords:** nurses, spirituality, spiritual care

## Abstract

The purpose of this study was to explore factors that influence nurses’ beliefs about offering spiritual care. Study Design: A mixed-method study design, incorporating both quantitative (questionnaire) and qualitative research, was used for this study (focus group discussion). Methods: The questionnaires were completed by a convenience sample of nurses and their assistants working in two public hospitals. These questionnaires included the Greek versions of the FACIT-Sp-12, SCIPS, NEO-FFI, and the Spiritual Climate Scale, as well as a specially designed questionnaire to gather demographic, socioeconomic, and professional information about the study population (SCS). Three nurses and two nursing assistants who worked in public hospitals and were chosen through purposive sampling made up the sample for the qualitative study. In utilizing inductive content analysis methodology, a qualitative analysis was carried out. Results: Greek nurses frequently offer spiritual care to their patients, primarily existential spiritual care. It was discovered that the spiritual climate, the nurses’ positive coherence, and their educational level all exert a favorable effect on total spiritual care. Three categories and seven subcategories were used to describe the participants’ prior experiences with spiritual care. Conclusions: Greek nurses frequently offer spiritual care to their patients, and both internal and external factors influence their attitudes in this regard.

## 1. Introduction

Spirituality is the fundamental factor that gives people’s lives purpose and meaning. For those who wish to live in peace with themselves and their surroundings, it is a necessary component. Everyone has an inner existence and a perspective that can be classified as spirituality, regardless of their religious affiliation [1].

Different definitions of spirituality have included philosophical, secular, and religious views. In this study, the definition of spirituality provided by the International Consensus Conference was adopted. According to this definition, spirituality is “a dynamic and intrinsic aspect of humanity through which persons seek ultimate meaning, purpose, and transcendence and experience relationship to self, family, others, community, society, nature, and the significant or sacred. Spirituality is expressed through beliefs, values, traditions, and practices” [2].

As one of the four facets of holistic care, spirituality includes a collection of principles, perspectives, and aspirations that link individuals to a higher being. It is a concept that transcends religious belief and strives to respond to queries concerning the meaning and purpose of life [3]. Spiritual care is a multidimensional notion that is described as caring behaviors that promote people’s spiritual health by achieving a balance between the physical, psychosocial, and spiritual components of life. Respect, empathy, attentive listening, and helping patients comprehend the sickness and its progress are all aspects of spiritual care [3].

### Background 

The body of knowledge in this field of spirituality and health has grown significantly over the last few decades, and the available data indicate that spiritual health is fundamental to both physical and mental health, bringing harmony and peace that can promote healthier lifestyles [4]. The choices made by patients and their families also seem to be significantly influenced by these beliefs [5]. Also, the World Health Organization (WHO) has acknowledged the significance of spirituality in wellness by incorporating “spirituality” as one of the critical elements in redefining “health”. In 1998, the WHO specifically highlighted spirituality as an aspect of health and backed the importance of patient satisfaction with unity and harmony in the domains of physical, social, mental, and spiritual health [5]. Most diseases currently take a long time to cure and recover from, and patients must deal with both the disease’s pain and the demands of family and society [6]. Nurses have to offer this care in a way that respects the patient’s spiritual beliefs, values, rights, and practices. Several health and social care professionals who engage with patients concur that spiritual care ought to be a crucial component of their services [7]. This is consistent with global nursing trends that emphasize a holistic approach to nursing care that considers all dimensions of a person, including spiritual care. If spiritual demands (such as the desire for love, hope, courage, meaning in life, purpose, and the trust and comprehension of others) and spiritual discomfort (negative emotions such as anxiety) do not receive relief and fulfillment, they might affect patients’ physical condition and the emotional side effects of treatment, leading to mental suffering. Assessing and addressing patients’ spiritual needs in close cooperation with other members of a multidisciplinary treatment team are the aims of spiritual care. To meet patients’ spiritual needs with spiritual care, it is critical to determine what services patients are not receiving. Issues of spirituality and its nature in nursing are connected to the notion of competency in spiritual care, which is described as the expertise, skills, and dispositions necessary to provide spiritual care [8]. In addition, improving nurses’ ability to provide spiritual care can increase not only patients’ satisfaction, but also the nurses’ well-being [9]. However, nurses still claim to be unaware of what spirituality is and to be ill-equipped to provide spiritual care in clinical practice, and the role of spirituality in patient treatment and care planning is sometimes disregarded [10]. 

Researchers have provided some preliminary findings regarding the impact of various factors linked to the frequency and competence of spiritual care provided by nurses. These factors include the demographic characteristics of nurses, their educational training background, self-perception, and attitude toward spirituality and spiritual care practice [10]. Regarding demographic variables, except prior hospitalization experience, there has been no significant correlation observed between competence to provide spiritual care and other factors including age, sex, marital status, and work experience [11]. In addition, a recent cross-sectional study that explored the factors influencing the attitudes of mental health nurses toward spiritual care highlighted both extrinsic factors, such as postgraduate education, working experience, participation in palliative care education programs, and spiritual care experience, as well as intrinsic factors like personality traits (e.g., Extraversion, Openness/Intellect, Conscientiousness, and Agreeableness), which were associated with greater awareness and inclination toward providing spiritual care [12].

Other factors that have been found to influence the capacity of nurses to deliver spiritual care encompass time constraints, worry regarding the patient’s response to their efforts to help with spiritual matters, and cultural and religious variances. A perceived deficiency in spiritual care skills, inadequate preparation, and an absence of confidence may also be involved [13]. 

Individual comfort levels and inadequate planning have also been found to be obstacles to providing spiritual care. Some nurses have anxiety when discussing spirituality with patients, particularly those who lack confidence in this area or are experiencing personal spiritual conflicts. Nonetheless, many nurses express uneasiness when discussing spirituality since it might be perceived as being too personal, intrusive, or divisive [10].

In summary, the nursing literature demonstrates that spirituality and spiritual care are integral components of patient care. However, there are still uncertainties regarding the meaning and borders of spiritual care within the nursing discipline [14]. Regarding this topic, relatively few studies have been conducted in Greece.

Based on the above, the current study’s goal was to explore the factors, both intrinsic and extrinsic, that shape nurses’ attitudes toward the provision of spiritual care. 

The present study was guided by the following research questions:

How do nurses and their assistants view spirituality and the provision of spiritual care?

What is the correlation between the provision of spiritual care by nurses and their assistants and their personal (demographic and socio-economic) and professional characteristics?

What is the correlation between the provision of spiritual care by nurses and their assistants and external factors such as workplace conditions?

## 2. Materials and Methods

### 2.1. Design

A mixed methodological approach was used to create a more comprehensive picture of the studied issue and a stronger research result compared to that which would result from each method separately [15]. This approach involved combining quantitative (questionnaire) and qualitative (focus group discussion) research in the same study. The method of triangulating two sets of findings can improve comprehension and increase the reliability of conclusions [16]. By utilizing mixed methods, researchers can obtain rich data that would not be obtained using either method alone, bypassing the limitations of quantitative and qualitative approaches [15,16].

### 2.2. Sample and Procedure

This study’s sample consisted of nurses and nurse assistants from two big public, general hospitals located in the urban area of the 1st health district in Athens, Greece. The questionnaires were distributed by hand to 365 nurses of the participant hospitals, of which 298 (response rate 81%) returned the questionnaires. Finally, 275 nurses and assistant nurses provided the full data used for the analysis. Hospital level and accessibility were factors that influenced the selection of these facilities. The sample was approached through convenience sampling. The decision to use this sampling technique was based on limitations in time, access, and finances, since it is simple, inexpensive, and requires less time than other sample techniques [17].

The inclusion criteria were that the participants were nurses and nurse assistants, male and female, with at least one year of experience, and not suffering from a condition that could affect their mental functions.

Participants were recruited through face-to-face invitations. A researcher visited the nurses during their break to discuss the study and invited them to participate. The distribution of the questionnaires was carried out during free time to prevent disruption at work, to provide all the information required for the study’s purpose, to provide the necessary instructions, and to clarify any questions.

Accordingly, the sample of this qualitative study consisted of three nurses and two nursing assistants who also participated in the quantitative study and who were selected through purposive sampling, according to their willingness to participate and their availability for the focus group. The criteria for inclusion were based on nurses and nursing assistants who provided direct patient care, with at least one year of experience. The qualitative approach used was a focus group aimed at in-depth exploration of the participants’ subjective views of the concepts of spirituality and spiritual care as well as the factors influencing their provision. An interview guide was created for the study.

### 2.3. Measures

The following tools were used to collect the data:

(a) A specially designed questionnaire was used to collect demographic (gender, age, place of permanent residence), socio-economic (marital status, cohabitation, work, education level, employment status), and professional (e.g., professional experience, job position, job department) information about the study population.
(b) To assess spirituality, the Greek version of the Functional Assessment of Chronic Illness Therapy-Spiritual Well-Being 12 scale—(FACIT-Sp-12) [18] was used. The wider Functional Assessment of Chronic Illness Therapy (FACIT) measurement system, which rates multidimensional HRQoL, includes the FACIT-Sp-12. The scale has shown strong reliability and validity among sizable samples of people from various ethnic backgrounds. Apart from two negatively worded questions (4 and 8) that were reverse-coded, the scale has 12 items formatted on a five-point Likert scale, with 0 denoting “hardly at all” and 4 denoting “very much”. Answers to self-reported questions relate to the 7-day recall period [19]. Meaning (items 2, 3, 5, 8), peace (items 1, 4, 6, 7), and faith (items 9, 10, 11, 12) are the three subdimensions of the scale. The dimensions’ scores range from 0 to 16, while the overall score ranges from 0 to 48. The responses to various elements are added to determine the score. Higher results imply greater spiritual well-being. A low level is defined as being less than 24, a medium level as being between 24 and 35, and a high level as being greater than 36 [20].
(c) The Spiritual Care Intervention and Spiritual Well-Being Questionnaire (SCIPS), a 17-item self-report scale developed by Musa and Pevalin to determine how often Arab Muslim nurses provide different forms of spiritual care intervention to their patients [21,22], was used. The questionnaire contains two subscales, a religious (RSCIPS, religious convictions, perceptions regarding God, relationship with God, and religious practices) and existential subscale (ESCIPS, relationship with self and others, such as cultivating a spirit of love and kindness, hope, preserving meaning and purpose, respect, and attentive listening). The religious subscale consists of eight items and the existential of nine items [21,22]. On a four-point Likert scale, responses range from “never” to “rarely” to “occasionally” to “often” (4). Higher scores indicate a stronger supply of spiritual care interventions. The aggregate SCIPS score is calculated by adding the replies to each of the 17 elements, with a possible score range of 17 to 68 [19,20]. The Greek translation and adaptation of the scale have been carried out by Fradelos et al. (2020) and have satisfactory psychometric qualities [23].
(d) The questionnaire NEO Five-Factor Inventory (NEO-FFI) [24], a popular self-report personality trait inventory with 12 items, each on neuroticism, extraversion, openness, agreeableness, and conscientiousness, that have been proven to correlate with the five-factor model of personality, was used. The NEO-FFI is mostly recommended for situations where providing the full instrument is not practical. It consists of 60 questions chosen from the NEO PI-R to measure the five domains [25]. On a Likert-based scale, each item is scored between 0 and 4, with 4 representing “strongly agree.” Twenty-eight of the 60 items have reversed wording [26]. After the appropriate items are reversed, the scale scores are calculated by adding the 12 items for each dimension. NEO-FFI is a widely used scale that measures the five dimensions of personality (personality traits): Agreeableness, Conscientiousness, Neuroticism, Extraversion, and Openness to Experience. The scale has been confirmed in numerous countries, including Greece, and has been translated into several languages [27].
(e) The Spiritual Climate Scale (SCS), the scale created by Doram et al. (2017) to evaluate the spiritual climate among health workers, includes four items, with the answers provided on a five-point Likert scale, ranging from ”disagree completely” (1) to “strongly agree” (5) [28]. The average of the four questions is used to calculate scores, which are then multiplied by 25 after being subtracted from the estimated mean by 1 [29]. For this scale, a total score from 0 to 100 can be calculated—the more positive the spiritual climate, the higher the score. The original version calculated Cronbach’s alpha as 0.863, indicating that it had good psychometric qualities [28]. The scale has been translated and validated to be used for the Greek population [30].

Qualitative data were collected with a semi-structured interview guide with questions derived from the literature review [31,32]. The interview guide consisted of two opening icebreaking questions regarding nursing experience followed by questions such as what your perception of spirituality and spiritual care is and what are the factors that influence spiritual care provision. The focus group interview lasted approximately 95 min. This duration allowed for thorough discussion and exploration of the topic. The discussion with the focus group was audio recorded, and afterward, the audio recording was transcribed verbatim. The transcription was performed by the principal investigator to ensure accuracy and reliability in transcribing the recordings. The transcripts were then analyzed using a coding process. Coding was conducted manually as follows. First, the transcript was reviewed line by line to identify significant statements and ideas. The initial codes were generated without any preconceived categories, allowing for flexibility and openness to emerging themes. Codes were then grouped and organized into broader categories and themes based on their similarities and relationships. Then, another member of the research team reviewed the qualitative data to confirm the analysis. 

### 2.4. Ethical Considerations

This study was approved by the Ethics Committee of the University of Peloponnese (2112/09022021) and by the committee of Hippokratio Hospital (ES.18ο24092019) and Sotiria Hospital (9633/26062019). All nurses were provided detailed and complete information about the purpose and objective of the study. Participants were also fully informed of their rights. A written informed consent was signed by each participant to declare their understanding and their voluntary participation in the study before completing the questionnaire. The questionnaires were anonymous to ensure absolute respect for the privacy and confidentiality of the nurses’ data. Participants were provided a white envelope to place their completed forms in before sealing them and sending them back to the researchers. All completed questionnaires were stored in a locked cabinet, to which only the researchers had access until the data collection was over. Participants were not incentivized to participate in the present study.

### 2.5. Statistical Analysis of Quantitative Data

The statistical package for social sciences (SPSS) software v.25 was used to carry out the statistical analysis. Quantitative variables were reported with descriptive statistics, while qualitative variables (gender, marital status, work, scale categories, and the remaining categorical variables) were reported with frequencies. The normality of the quantitative variables was checked using the Kolmogorov–Smirnov statistical test. A comparison of the total scores of the scales with variable differences was performed using the Student’s *t*-test and one-way ANOVA statistical tests. To examine the relationships between the scales, Pearson’s correlation coefficient was also used, while to investigate the factors related to the provision of spiritual care and nurses’ caring behaviors, the example of linear regression with a stepwise integration of variables was used. The level of statistical significance was set at *p* < 0.05. 

### 2.6. Analysis of Qualitative Data 

The analysis of the qualitative data resulting from the transcription of the interviews was conducted according to the principles of the qualitative methodology of inductive content analysis [33].

## 3. Results

According to Table 1, 85.4% of the sample was female. Their age was an average value of 43.6 years, and 63.6% were married. Regarding their educational level, 24.7% were graduates of secondary education, and 44.4% were graduates of higher education. The employees’ seniority had an average value of 20.1 years. Most of them did not hold any institutionalized position of responsibility at work (90.8%). The study nurses who held positions of responsibility were also included as these nurses also have contact with the patients and enter the patient’s room, and if the conditions are favorable, they may provide spiritual care.

The mean value for the spiritual care scale was found to be 47.20 (±9.13); for existential care, 29.40 (±4.76); and for religious care, 17.80 (±5.68). The perceived spiritual climate was found to be 58.13 (±23.21). Regarding nurses’ spirituality, the total score was found to be 34.39 (±7.30). Detailed descriptive measures for the research tools are presented in Table 2.

From a bivariate analysis on the effect of participants’ demographic and occupational characteristics on the provision of spiritual care, only the following was observed: work departments appeared to have a statistically significant effect on the provision of spiritual care, with participants working in outpatient departments having a lower score on providing spiritual care. The detailed results are presented in Table 3.

Concerning predictors influencing the provision of spiritual care by nurses, to identify the factors that influence the provision of spiritual care by nurses, the examplar of multiple linear regression was used. The Spirituality Scale, Spiritual Climate Scale, Personality Questionnaire, and nurses’ characteristics were used as independent variables, while spiritual care was used as a dependent variable in the multiple linear regression (stepwise method). From the regression analysis, it was found that the spiritual climate, the nurses’ positive coherence, their agreeableness, their educational level, and the dimension of meaning in the nurses’ spirituality have a positive effect on overall spiritual care. In detail, the results for the spiritual care of nurses, both in total and at the level of the subscales, are presented in Table 4.

### 3.1. Presentation of Qualitative Research Results

A total of five nurses participated in the qualitative study (Table 5). The experiences reported by the participants in the qualitative part of this study are varied, which is mainly attributed to the diversity of the work departments but also to the uniqueness of the individual and the motivations that drive them. Some became involved in nursing consciously, either out of the need to provide or for purely livelihood reasons, while others found themselves in nursing by accident, but through their professional path, they seemed to manage to derive pleasure from it and developed both professionally and personally. One theme accurately describes their experience of spiritual care so far, and that is “The Long Journey of Spiritual Care”. Participants, through a journey through the nursing profession, go through several stages until they can provide spiritual care to their patients and derive pleasure and personal growth from it. A total of three categories emerged from the analysis of the results and seven subcategories (Table 6).

### 3.2. Category 1: Discovering Spiritual Care

This category consists of three subcategories: (a) spiritual care as a calling, (b) developing self-confidence in the workplace, and (c) developing communication skills.

(a) Spiritual care as a calling.

Nurses recognize the significance of spirituality in maintaining good health and promoting healing. Assessing and addressing patients’ spiritual needs are parts of holistic nursing. In addition, they recognize the significance of spirituality in maintaining good health and promoting healing for them. Spiritual care starts with compassionate relationships.

“*having a close connection with the patient, having faith in what you do and to generally believing in the patient and in all nursing as an ideology and loving the patient…*”

N1

“*…I consider spirituality as a gift, which few people have. I think that most nurses have it. Because they can understand their fellow man…*”

N5

(b) Developing self-confidence in the workplace.

During the focus group, the importance of self-confidence in clinical practice spiritual care was highlighted. Participants stated that holistic assessments and patient holistic care are parts of their work. Nurses use critical thinking and personalized healthcare plans to provide holistic care to their patients.

“*I familiarize myself with them, I try to “fit” them in the atmosphere that is, in addition to the personal, the physical care that I provide, I personally also provide social care and social commentary and social history and the embodiment of many things that flow from there.*”

N1

“*…the experience, and also very important for me is the critical thinking of the nurse. The nurse, a key part in my opinion, must have critical thinking. Each patient must be provided with a different type of spiritual help both in terms of time and quality. So they judge and know how to handle it…*”

N3

(c) Developing communication skills and empathy.

One other major topic that emerged during the focus group was communication skills and empathy. Effective communication takes place when nurses listen closely to patients, show them respect and decency, and understandably provide information.

“*In Nursing in everyday life you may be able to use it… not always and not for all patients. In patients where you can see results, you can use it. When there is communication. When they want it themselves. When you also understand that someone wants it. Some patients wish to communicate with you and some patients do not wish to communicate. To those who wish to, and you must realize it, empathize with it, you can very nicely and very much offer above all…*”

N1

“*Yes, I want to say that we must know communication well, the art of communication. That is to be good listeners, good speakers, and communication skills. It’s a key part.*”

N4

### 3.3. Category 2: Overcoming Cultural Barriers

This category consists of two subcategories: (a) spiritual care as a common cultural value and (b) spiritual care against strictly oriented nursing work.

(a) Spiritual care as a common cultural value.

To interact with diverse people with diverse beliefs, the nurse needs to maintain a comprehensive awareness of spirituality. Becoming familiar with patient’s cultures helps them understand the knowledge, beliefs, assumptions, and values of their patients. Spirituality is a profoundly innate sense of interconnectedness with the world that is not always consciously expressed. Looking at the “big picture”, which transcends culture, religion, or the condition of health, is the most crucial factor, and that makes the difference.

“*…that spirituality has to do with how we perceive the general spirit of the patient before us, *i.e.,* their ideas, their psychosocial situation, their religious beliefs, their cultural and cultural beliefs, their ideas, and being able to make them feel comfortable and able to express themselves and communicate with us.*”

N3

“*That’s where I start. Social history, social care, support, communication, from social fishing, health fishing and all that comes. I familiarize myself with them, I try to “set” them in the atmosphere that is, in addition to the personal, physical care that I provide, I personally, I personally also provide social care and social commentary and social history and the embodiment of many things…*”

N1

“*If the patient and the nurse are in the mood, of course. And this greatly improves their quality of life, it relieves them of the stress that most of them have…*”

N2

“*… it has to do with understanding the general spirit of the patient before us, *i.e.,* their ideas, their psychosocial situation, their religious beliefs, their cultural and cultural beliefs, their ideas, thus being able to make them feel comfortable and be able to express themselves and communicate with us. So, I think it is a very basic characteristic that the nurse should have and if they can develop it, there is better communication based on these characteristics…*”

N3

(b) Spiritual care versus rigidly prescribed nursing work.

Nurses describe the obstacles that they are facing as they try to provide spiritual care and not only rigidly prescribed nursing work. These obstacles, both human and environmental, are lack of time, educational preparation, and lack of confidence; nevertheless, the mutual understanding and shared culture in the ward between colleagues seem to be the basis for change.

“*It depends purely on the mood and conscientiousness of the nurse. Net! And not only from the working conditions and from the other nurses. Because in a clinic when you go and treat the patients spiritually, there are colleagues who say you are spoiling the market…..*”

N1

“*…. that it doesn’t help at all when there is one nurse for 40 people. What will be the help they can provide spiritually to the sick person? None to minimal. So, it is an inhibitory factor. When there is no staff to do the basics, to do the nursing, how can we deal with their mental state and help them. Helpfully, I could say, the experience that each nurse has works….*”

N3

“*The environment. The colleagues… There are colleagues, some colleagues have told me, that you are “ruining the market”, “don’t be so busy”, because if they come again and again, they will demand more from us.*”

N1

### 3.4. Category 3: Satisfaction from Compassion and Spiritual Development

This category includes two subcategories: (a) satisfaction with holistic nursing care and (b) personal/professional development and self-care.

(a) Satisfaction from compassion and spiritual development.

Helping patients and providing spiritual care are considered satisfying aspects of their work, and nurses consider that these contributions reward them spiritually as well. In addition, they talk about the satisfaction that they are receiving from being present and providing compassionate care.

“*Yes, and I really liked it, we had a great time. The sick people… We were quite attached to the sick, because they stayed there for a long time”……… but you could see that you gave them a lot of courage, that you comforted them, that they got strength from you. Especially when they were leaving and then coming back to see us. Okay, you said, that’s it! That is a great feeling! Having a sick person returning to the hospital to see you, for example after a year…*”

N2

“*…[…] that is, the person is lost at that moment, and you have to be there to comfort them in any way you can, to tell them something good, to be able to calm them down, to tell them that everything will be fine…[ …] The diseases are chronic, for sure. Because the patients come again and again, you try to support them. Because their body is slowly giving up, let’s say, and you are trying to strengthen them spiritually. Comfort them…*”

N5

(b) Personal/professional development and self-care.

Some nurses did not anticipate developing close bonds with patients and their families, but daily encounters with life and death made them aware of their own values, beliefs, and moral compass. They start to appreciate the little things every day and feel more human. Moreover, they feel confident because they know how to help their family, and they recognize new aspects of personal and professional development.

“*I keep discovering aspects of myself… I discovered another at Christmas, on New Year’s, and another at Easter… because they were completely alone and needed the companionship that I provided. The neighbor, the granddaughter, the friend, and the nurse…[…] And there I discover many aspects of myself, the last aspects of myself […]. There I discovered the last aspect of myself, which was that I liked nursing. I felt lucky to be able to endure nursing because I must repeat, I was not disgusted, I was not afraid, I could offer, conscientiously, I felt good because I did well in everything I could do, and I felt no regrets, and continued my further course. And then I discovered that I would like, both for development and for financial benefit, to take the exams and advance to a higher level… and all these years, every year, every year, every year I am getting used to it better and I can offer more.*”

N1

“*I liked my job as an object because I offer to the patient, I help them and whatever they need, they tell me, and I try to do it. Of course, nursing helped me for my family, too…*”

N5

## 4. Discussion

The purpose of the present study was to investigate the factors, both intrinsic and extrinsic, that shape nurses’ attitudes toward the provision of spiritual care. 

The findings of the quantitative study indicate that the mean value for the spiritual care scale was 47.20 (±9.13), suggesting that nurses and nurse assistants provided spiritual care interventions infrequently. Furthermore, our analysis revealed that the mean value for existential care was 29.40 (±4.76), whereas for religious care, it was 17.80 (±5.68). These results indicate that nurses administered religious spiritual care interventions to their patients less frequently than existential spiritual care interventions. This result aligns with the findings of Musa (2017) [21] and Albaqawi et al. (2019) [34], which demonstrated that existential interventions have been commonly used by nurses as part of spiritual care.

The current investigation revealed a positive spiritual climate in the workplace, with a perceived spiritual climate of 58.13 (±23.21). Additionally, our findings indicate that Greek nurses possess a high level of spiritual well-being, as evidenced by a total score of 34.39 (±7.30) on the nurses’ spirituality assessment. 

Our study revealed a statistically significant relationship between work departments and the provision of spiritual care. Specifically, individuals working in outpatient departments had a lower score in delivering spiritual care. Albaqawi (2019) observed an important relationship between the type of hospital and the provision of spiritual care interventions. They claim that these findings are consistent with earlier empirical evidence, which indicate that the spiritual climate differs significantly among various hospitals. Various aspects of spirituality content, such as belief in a higher power, moral principles, faith, values, love, and relationships, contribute to an individual’s uniqueness. These characteristics probably offer a potential explanation for the observed variances. Furthermore, different kinds of hospitals have different organizational cultures, and diverse leadership styles have an impact on the sense of workplace spirituality [34]. 

Workplace spirituality in the healthcare system involves searching for a meaningful and purposeful existence, fostering positive connections with colleagues, clients, and families, and maintaining coherent individual and organizational beliefs and values. The term “spiritual climate” refers to the collective awareness and engagement of employees with spirituality in the workplace [35]. In our study, the spiritual environment was found to have a good influence on the overall provision of spiritual care. The result is consistent with earlier findings [36].

The mean values of SCIPS, RSCIPS, and ESCIPS in the current study were 47.20 ± 9.13, 17.80 ± 5.68, and 29.40 ± 4.76, respectively, with these values corresponding to the theoretical mean values. So, we can claim that patients receiving spiritual care from Greek nurses are frequently administered existential spiritual care. In a study on psychometric properties of the scale [37], as well as other studies carried out in comparable cultural contexts [5,38], nurses scored higher on the SCIP scale when compared to our results. This finding can be explained through cultural factors as well as by how nurses perceive providing spiritual care. Also, even though the ESCIPS value is satisfactory, it is important to note that in all these studies, nurses reported a lower score on the subscale of RSCIP. Our findings agree with the above studies. Perhaps the discrepancy in values can be attributed to nurses’ expectations that these needs will be met by experts like priests and religious leaders [21,39].

In the present literature, the field of clinical practice has been proposed as a factor impacting nurses’ comprehension of spirituality and spiritual care. According to a recent study [40], the frequency of spiritual care varies significantly depending on the context. In other words, nurses who specialize in palliative care provide spiritual support more than nurses who work primarily in acute or hospital settings [41]. 

Work experience was not related to the provision of spiritual care in the current study. Similar to the current study are the results of other authors [42], where there was no significant relationship between sociodemographic characteristics and spiritual care competence, including nurses’ experience.

The results of the literature are contradictory concerning the participant demographics and how they affect the provision of spiritual care. For instance, Melhem et al. (2016) discovered that there are significant differences in spiritual care views between women and men [43]. However, a subsequent study found no connection between a nurse’s spiritual care competency and demographic factors [44]. Similarly, it was determined that the participants’ details, such as gender and educational attainment, do not statistically affect the provision of spiritual care significantly.

The spiritual climate was found to have a favorable impact on overall spiritual care, which is in line with a recent study’s findings [34]. A healthy spiritual atmosphere fosters a strong sense of purpose and greater life fulfillment in a clinical practice setting [34]. Finally, in contrast to a previous study [45], the present one found that nurses’ educational background is related to their overall spiritual care. 

The qualitative study highlighted that spiritual care is a nurse’s journey, a calling, and a long road of personal work and growth. For its provision, experience, familiarity with patients, the development of self-confidence in the workplace, and communication skills and empathy emerged as particularly important factors, while the strictly predetermined nursing work, the lack of staff and support, and the general environment of the hospital were mentioned among the obstacles to its provision. The responses of the participants in this study also demonstrated the significant contributions of spirituality and the provision of spiritual care to their professional, personal, and spiritual development.

According to the responses of the participants, providing spiritual care is both a calling and a gift. Florence Nightingale also emphasized nursing as a calling—a vocation, stating, “but more than that, a nurse must be a religious and devoted woman. She must have respect for her calling because the precious gift of life from God is often placed literally in her hands” [46]. Nurses gain confidence in assessing patients’ spirituality, recording necessary information about spiritual needs, and recognizing issues that necessitate providing spiritual care by speaking with their colleagues [47]. The above is reflected in the statements of two of our study’s participants. In another study, a participant similarly illustrates the nurse–patient relationship with the following phrase: “I think to provide spiritual care, you need to form a bond or a rapport with the patient, I think that it is no different than saying the kind of rapport that you might have with someone you are attracted to” [48]. The responses of the participants in this study also demonstrated the particular importance of developing communication skills when providing spiritual care, as well as empathy. This finding corroborates with that of a recent study, according to which the caring style and communication skills of nurses can facilitate the therapeutic relationship [49]. 

Our study’s participants viewed spiritual care as a shared cultural value. Nurses with a variety of spiritual perceptions are requested to express their views, explore each other’s beliefs together, and aid one another in a holistic spiritual perspective on nursing practice. In particular, the spirituality of individuals is pertinent to their culture because it may be entirely affected by cultural standards, be in contradiction with them, or be influenced by cultural standards and personal life events. A comprehensive evaluation of the patient’s spirituality, focusing on how much the patient’s cultural background has influenced their spirituality, is also necessary. This evaluation allows nurses to adjust the nursing care plan recommended in the literature and customize it to the patient’s cultural and personal spiritual beliefs [50].

Regarding the barriers to spiritual care, in the international literature, among the obstacles that nurses may face in their effort to provide spiritual care are, among others, a lack of time, a lack of support, nurse discomfort [51], a lack of human resources, and an increased workload [52]. The barriers mentioned by the participating nurses in the present study are similar.

Florence Nightingale [1820–1910], the creator of modern secular nursing, regarded spirituality as an integral aspect of human flourishing and as the deepest and most potent healing resource available to the individual, with significant nursing implications [53]. The above is reflected in the statements of the participants in our study. According to the participants in this study, the contribution of spiritual care and spirituality to their professional and personal development is important. This finding is consistent with the literature, which has demonstrated that spiritual care enhances people’s spiritual performance, enhances their sense of integrity and excellence, and improves interpersonal relationships [54].

There were several limitations in this study. For instance, our sample only included nurses and nursing assistants working in public hospitals in major cities, so it might not be typical of other provincial or non-provincial areas of the nation or private hospitals. The small sample size of only 275 participants is a further limitation since it has been shown that very small samples compromise a study’s internal and external validity [55]. A limited sample size may limit the generalizability of the findings, thereby limiting the work’s applicability [45]. One more major limitation of the present study is that only one focus group was contacted with the five participants. Future research should aim to replicate the existing data across the nation using a larger, more representative sample as well as more focus groups with more participants. 

On the other hand, one of the advantages of the current study is the variety of data-gathering techniques employed to extract pertinent information, which helped to increase the study’s reliability.

## 5. Conclusions

The purpose of this study was to investigate the factors associated with nurses’ attitudes toward the provision of spiritual care. The provision of spiritual care is an important and integral element of holistic care, with positive results and benefits for both nurses and patients. The qualitative study highlighted that spiritual care is a nurse’s journey, a calling, and a long road of personal work and growth. For its provision, experience, familiarity with patients, the development of self-confidence in the workplace, and communication skills and empathy emerged as particularly important factors, while the strictly predetermined nursing work, the lack of staff and support, and the general environment of the hospital were mentioned among the obstacles to its provision. The responses of the participants in this study also demonstrated the significant contributions of spirituality and the provision of spiritual care to their professional, personal, and spiritual development. Also, it was found that Greek nurses often provide spiritual care to their patients with a positive spiritual climate, coherence, and tolerance, while the high educational level of the nurses has a positive effect on their provision.

Incorporating spiritual care into nursing practice has the potential to enhance patient-centered care by addressing the comprehensive needs of individuals. Policymakers, healthcare providers, and nursing services have a critical duty to establish structured norms, methods, and training on the provision of spiritual care with the aid of government organizations. To ensure that nurses and other health workers can provide spiritual care, it is also crucial to successfully train and educate them. This would enhance working surroundings and circumstances and enable nurses to deliver higher-quality healthcare. The design of appropriate strategies and interventions that will help to strengthen the capacity of nurses to provide this type of care as well as encourage spiritual practice to promote the spiritual well-being of both nurses and patients is dependent on the recognition of the factors that influence nurses’ attitudes toward the provision of spiritual care. This study also advances the field of nursing education, as it is necessary to incorporate spiritual care with humanistic training to develop certain capacities and competencies for practicing holistic nursing.

## Figures and Tables

**Table 1:** Characteristics of the sample.

	N (%)
Gender	
Female	235 (85.4)
Male	40 (14.5)
Age	43.6 ± 8.5
Marital status	
Unmarried	75 (27.2)
Married	175 (63.6)
Widow/widower/divorced	23 (8.3)
Educational level	
High school	68 (24.7)
Tertiary	122 (44.4)
Post-graduate studies	80 (29.0)
Ph.D.	8 (2.9)
Specialization	
Yes	131 (47.6)
No	144 (52.4)
Working experience as a nurse (years)	20.1 ± 9.5
Department	
Outpatient clinics	31 (11.2)
Pathology	126 (45.8)
Surgical	44 (16)
I.C.U.	55 (20)
Other	19 (6.9)
Work Position	
Nurse assistant	69 (25)
Nurse	181 (65.8)
Chief	22 (8)
Department head	3 (1.09)

**Table 2:** Descriptive measures and reliability of survey tools.

	Mean ± SD	Cronbach’s a
Spiritual care scale		
Religious care	17.80 ± 5.68	0.850
Existential care	29.40 ± 4.76	0.854
Total spiritual care	47.20 ± 9.13	0.887
Spirituality		
Total spirituality	34.39 ± 7.30	0.789
Meaning	14.21 ± 2.09	0.644
Peace	9.89 ± 3.12	0.733
Faith	10.38 ± 423	0.888
Personality traits		
Neuroticism	32.89 ± 5.49	0.731
Extroversion	41.90 ± 5.54	0.750
Openness to Experience	39.21 ± 5.14	0.831
Agreeableness	45.45 ± 5.61	0.835
Conscientiousness	49.23 ± 6.45	0.759
Spiritual climate	58.13 ± 23.21	0.920

SD: Standard Deviation.

**Table 3:** Analysis of variance by one factor.

	Outpatients’ Department (1)	Pathology (2)	Surgical (3)	I.C.U (4)	Other (5)	
Religious care	13.63± 5.4	19.0 ± 5.1	17.9 ± 5.6	18.1 ± 5.9	15.9 ± 5.3	6.781 ***
Existential care	25.06 ± 5.7	29.8 ± 4.6	30.1 ± 4.3	29.8 ± 4.2	29.8 ± 3.9	7.677 ***
Total spiritual care	38.70 ± 9.7	48.9 ± 8.3	48.0 ± 8.6	48.0 ± 9.0	45.8 ± 7.9	8.855 ***

*** *p* < 0.001.

**Table 4:** Multiple linear regression (stepwise method) with dependent variable spiritual care and independent variables Spirituality Scale, spiritual climate, personality traits and caring behaviors, and characteristics of nurses (N = 275).

Spiritual Care Scale	Factors	B	SE	95% CI	*p*-Value
Total spiritual care	Spiritual climate	0.091	0.021	0.049–0.133	<0.001
	Agreeableness	0.237	0.091	0.059–0.416	0.009
	Educational level				
	Secondary (control group)	0			
	Tertiary	1.334	0.604	0.146–2.523	0.028
	Meaning	0.499	0.233	0.040–0.958	0.033
	Adjusted R^2^ = 27.2%. F = 21,449 *p* < 0.001				
Existential care	Agreeableness	0.206	0.044	0.120–0.292	<0.001
	Department of occupation				
	Outpatient department (control group)				
	Hospitalized patients	0.583	0.203	0.183–0.983	0.004
	Spiritual climate	0.029	0.011	0.008–0.050	<0.001
	Sex				
	Male (control group)				
	Female	1.741	0.705	0.353–3.129	0.014
	Educational level				
	Secondary (control group)				
	Tertiary	0.601	0.303	0.004–1.197	0.048
	Adjusted R^2^ = 34.4% F = 21,518 *p* < 0.001				
Religious care	Spiritual climate	0.060	0.014	0.032–0.089	<0.001
	Total spirituality	0.101	0.046	0.010–0.019	0.029
	Adjusted R^2^ = 17%. F = 17,145 *p* < 0.001				

B = Regression Coefficient, R^2^ = Coefficient of Determination, SE = Standard Error, CI = Confidence Interval.

**Table 5:** Characteristics of the sample of qualitative research.

Code	Gender	Age	Work Position	Education	Working Experience	Department
N1	Female	47	Nurse	BSc	20	Surgical
N2	Female	40	Nurse	BSc	19	Surgical
N3	Female	40	Nurse Assistant	Diploma	16	Medical
N4	Female	45	Nurse	BSc	13	Outpatient
N5	Female	39	Nurse Assistant	Diploma	17	Medical

**Table 6:** Results of the qualitative research.

Theme	Categories	Subcategories
The Long Journey of Spiritual Care	Discovering spiritual care	Spiritual care as a calling The development of self-confidence in the workplace Development of communication skills
	Overcoming cultural barriers	Spiritual care as a common cultural value Spiritual care against strictly oriented nursing work
	Compassion satisfaction and spiritual growth	Satisfaction with holistic nursing Personal/professional development and self-care

## Data Availability

Data supporting this study are available from the corresponding author upon reasonable request.
